# Exploring emotional wellbeing in the perinatal period: A qualitative study in Australia

**DOI:** 10.1177/22799036251395270

**Published:** 2025-11-13

**Authors:** Lesley Pascuzzi, Karen Heslop, Helen Skouteris, Zoe Bradfield

**Affiliations:** 1 School of Nursing, Faculty of Health Sciences, Curtin University, Perth, WA, Australia; 2 Department of Nursing and Midwifery Education and Research, King Edward Memorial Hospital, Women’s and Newborn’s Health Service, Perth, WA, Australia; 3 Health and Social Care Unit, School of Public Health and Preventive Medicine, Monash University, Melbourne, VIC, Australia

**Keywords:** perinatal mental health, maternal mental health, emotional wellbeing, qualitative descriptive design, mental health promotion

## Abstract

**Background:**

The transition to motherhood is well-known as a significant event in the life span and improved mental health and emotional wellbeing are known to improve maternal and neonatal outcomes. Maternal mental health is reported to positively impact pregnancy health outcomes, elevating levels of public health overall. However, misalignment exists between women’s perspectives and policy frameworks that govern healthcare. This study provides an opportunity to explore perspectives for the first time in Australia. The objective of the study was to explore women’s knowledge of recognizing emotional wellbeing and experiences of focused promotion in the perinatal period.

**Design and methods:**

A qualitative descriptive approach was adopted. Twenty-one women who were planning pregnancy, currently pregnant or raising children under 10 were recruited. Semi-structured interviews and focus groups were conducted and data analyzed using inductive thematic analysis.

**Results:**

Five themes were generated: Emotional Wellbeing is something we feel; Emotional Wellbeing is a state of being; The Impact of Environment; What Women Want and Perinatal Mental Health Literacy.

**Conclusions:**

Australian women define emotional wellbeing as the absence of illness, multidimensional and heavily shaped by the influence of social media, interpersonal relationships, and antenatal midwifery models of care. Findings show improving perinatal mental health literacy among women, families, and maternity care providers may enable earlier recognition and support for emotional wellbeing in the transition to motherhood. Further research is warranted to target knowledge and awareness of emotional wellbeing to support the development of a mentally healthy vocabulary for pregnant women and mothers.

## Introduction

The World Health Organization (WHO) states that there is “no health without mental health,”^1^ emphasizing that health is more than the absence of disease. Despite this holistic definition, Australian research continues to focus on identifying and treating perinatal mental illnesses to improve overall population health. Perinatal mental health improvements are commonly reported in terms of effective management, early identification or reduced risk of anxiety and depression^2^ aligning with priorities outlined in national perinatal mental health guidelines.^3^ This focus reflects the broader public health concern in Australia,^4^ where one in five women diagnosed with mental illness. Concsequently, clinical practice is centered on identifying risk factors or diagnosing conditions including anxiety and depression. Empirical research similarly emphasizes prevention as risk reduction, rather than promoting proactive wellbeing promotion for all women during this critical life stage. The differing nomenclature between prevention of mental illness and promotion of mental health extends worldwide where outcomes such as “reduced depression and anxiety” are frequently reported in research as indicators of mental health promotion.^5–7^ Furthermore, indicators for “perinatal mental health” to inform global framework cite categories including depression, anxiety, post-traumatic stress disorder, psychosis and adjustment disorders in the perinatal period.^8^

The perinatal period is defined as the time between pregnancy and 1 year post birth^9^ and is regarded as a significant event in a women’s life.^10^ During this time, women experience changes in health-related quality of life.^11^ Positive maternal mental health and wellbeing during pregnancy improve maternal and neonatal outcomes,^12,13^ throughout the first 2000 days of life^12,14^ and are associated with enduring positive effects on child development.^15^ The World Health Organization (WHO) proposes that maternal mental health is “a state of wellbeing in which a mother realizes her abilities, can cope with the normal stresses of life, can work productively and fruitfully, and can contribute to her community.”^16^ Mental health and wellbeing are inextricably linked, and the success of public health promotion directives requires effective translation and implementation to directly influence positive change in population health. A recent scoping review by Baldwin et al.^17^ explored definitive definitions of “maternal wellbeing” and revealed challenging differences between women’s perceptions of maternal wellbeing and the orientation of standard practices, policies and procedures to promote it during the perinatal period.^17^ Interrelated themes within the review center on the “sense of self” as a pivotal construct. A woman’s inner sense of self correlates with her mental health state and, during pregnancy, has been explored as a state of emotional wellbeing.^18^ Emotional wellbeing is included in definitions of perinatal mental health.^19^ Theoretical discussions of “maternal well-being”’ and “perinatal well-being”’ have included emotional wellbeing as a construct.^20^ However, there is no published research exploring Australian women’s perceptions of what it means to be emotionally well, making it difficult to operationalize the WHO definition. Across professional disciplines there is consensus that emotional wellbeing is related to a person’s overall sense of positivity about life.^18^ Baldwin and colleagues propose clearer articulation is needed for healthcare and professional practices to remain flexible and responsive to the individual needs of those receiving maternity care.

## Research aim

The aim of this study, therefore, was to explore the knowledge and experience Australian women have of identifying emotional wellbeing in the perinatal period to inform a needs assessment within a larger maternal mental health promotion project. The study was guided by the following research questions:

1. How do women mothers identify and conceptualize emotional wellbeing?
2. What are their perspectives of barriers and enablers to accessing opportunities to optimize emotional wellbeing?
3. What strategies do they identify as possible solutions to effectively support pregnant women and mothers toward optimal mental health and emotional wellbeing in the future?

## Methods

We employed a qualitative descriptive design to explore Australian women’s perceptions of identifying and optimizing emotional wellbeing during the perinatal period. Qualitative descriptive studies aim to describe phenomena rather than to explain it.^21^ This approach emphasizes low-inference interpretation and is appropriate for women’s health research.^22,23^ Participants were purposively sampled^24^ from those who responded to study promotions on social media platforms (Facebook, X, LinkedIn, Instagram) and relevant online community groups. Convenience and snowball sampling further helped recruit women planning pregnancy, currently pregnant, or parenting children under 10 from across Australia. Interested participants chose between one-on-one interviews or focus groups and received information and consent materials prior to participation.

### Data collection

Semi-structured, one on one interviews (*n* = 10) and qualitative focus group discussions (*n* = 2) with participants (*n* = 11) were conducted between May and June 2024 by PhD candidate (lead author) as part of a higher degree by research. Questions were informed by the conclusions of empirical research highlighting specific concepts of interest including a definition of emotional wellbeing,^19^ the current orientation of mental health screening in primary maternity care in Australia^25^ and findings of a scoping review.^17^ Semi-structured interviews were selected over in-depth interviews as a suitable way to extend knowledge^26^ of emotional wellbeing across the perinatal period. Focus groups are convened to discuss issues of mutual interest^27^ and are appropriate when researching mothers.^28^ Focus groups had 6 and 5 attendees respectively. Interview guides (Supplemental Files 1 and 2) were developed and included targeted open-ended questions. A pilot interview was hosted with one participant on Microsoft Teams, video recorded, and a transcript generated. This was reviewed and discussed between all authors and no changes made to the interview guide. Analysis of the pilot interview was not included in the results of this study. Engagement in interviews and focus groups was hosted on Microsoft Teams, lasted between 45- and 60-min duration, audio and/or video recorded and transcribed within Microsoft Teams. Sampling of participants was purposive^29^ until data sufficiency^30^ was achieved. Participants provided written consent and preferred meeting times were individually selected. On completion, all transcripts were downloaded from the audio/video recording were checked for accuracy by the first author (LP). No repeat interviews were carried out.

The first author has experience as a mother raising children in Australia and previous perinatal mental health qualifications and clinical experience interviewing to assess and diagnose perinatal mental health within primary care. When interviewing and facilitating focus group discussions, maintaining a research journal^31^ to document field notes including any pre-existing thoughts prior to data collection and attending regular supervision meetings with members of the research team to discuss the experience were adopted as strategies to reduce any personal bias affecting the quality of data collection and analysis. Co-author/supervision team consisted of an experienced midwifery leader and clinician researcher, mental health nurse academic/researcher and a developmental psychologist experienced in health promotion research and implementation science.

### Data analysis

Demographic data were collected from participants and are summarized in Table 1. Transcript data were analyzed using inductive thematic analysis (ITA). Thematic analysis^32^ is a six-phase method for identifying, analyzing, and reporting patterns (themes) within data. All transcripts were coded by lead author (LP) using NVivo 14 software. For validity, 20% of transcripts were coded by remaining authors. An inductive approach means the themes identified are strongly linked to the data themselves therefore was deemed to be an appropriate process of coding the data without trying to fit it into a pre-existing coding frame or match the data to the interviewer’s analytic preconceptions. All participants were involved in verification of the analysis of data. To establish validity^33^ and credibility,^34^ it is common to complete member checking in qualitative research. This was completed by the lead author, and all participants were presented with original data transcripts and researcher analysis of themes and subthemes by email communication. Participants were invited to review and respond. This process strengthens credibility by eliminating any misinterpretation during the analysis process.^33^

**Table 1.** Demographic characteristics of participants.

Demographic variable	Number (%)
Gender	
Female	21 (100%)
Age	
18–24	1 (5%)
25–34	5 (24%)
35–44	13 (62%)
45–54	2 (9%)
Location	
Western Australia	16 (76%)
New South Wales	2 (9.5%)
Victoria	2 (9.5%)
Northern Territory	1 (5%)
Stage of perinatal period	
Preconception	1 (5%)
Currently pregnant	1 (5%)
Raising children	19 (90%)
No. of children at home	
One	7 (33%)
Two	8 (38%)
Three or more	6 (29%)
Aboriginality	
Yes	1 (5%)
No	20 (95%)
Previous history of perinatal mental illness	
Yes	11 (52%)
No	10 (48%)
Any language other than English spoken at home	
No	21 (100%)

## Findings

Ten women participated in an interview. Of these, eight were mothers raising children, one identified as an Aboriginal Australian, one was currently pregnant with her first child, and one woman was actively planning pregnancy through in vitro fertilization. A total of 11 mothers attended focus groups, were aged between 18 and 54 and more than half had not previously been diagnosed with perinatal mental illness.

## Themes

**Figure 1.**
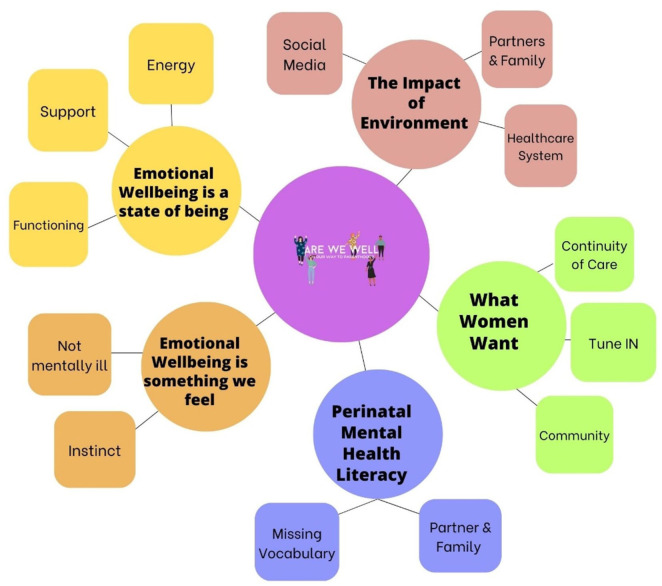
Themes and sub-themes.

Analysis of the data produced five main themes. Emotional Wellbeing is a state of being; Emotional Wellbeing is a something we feel; The impact of environment, What Women Want and Perinatal Mental Health Literacy represent the descriptions of women and mothers’ perspectives and experiences of emotional wellbeing across the perinatal period. A diagram of themes and sub-themes supported by concepts is presented in Figure 1. Additional quotes to support themes are presented in Supplemental Table 3 (Supplemental File 3).

## Theme 1: Emotional wellbeing is a state of “being”

Women conceptualized emotional wellbeing as a state of being when questioned on recognizing emotional wellbeing. Three sub-themes describe their perspectives: Energy, Support and Functioning within daily life.

## Energy

Most participants described emotional wellbeing related to having energy.

For me, certainly [emotional wellbeing] is like having energy and feeling motivated. . .being able to actually get things done, that is a really big indicator for me that my mental health is going well.

*Participant 10, woman planning pregnancy*

Prioritizing time to manage the demands of motherhood in relation to emotional wellbeing was described by a mother raising multiple children:

I’ve never had any problems with my own mental health, but I have had moments where I felt overwhelmed. Just tired, like I think every mum does. . . [Emotional wellbeing] is not all happiness. There will always be times when you’ve got to just be mindful of what you need. And then do it.

*Participant 8, mother raising children*

## Support

The uniqueness of the perinatal period and the need to “remember” was described by a participant in a focus group discussion, recalling the significance of having support during pregnancy around making birth choices:

Gee, that is interesting asking what it is [emotional wellbeing] because I have to kind of reflect now . . .I think it was about having supported conversations around what I wanted, what was important to me. . ..I guess I kind of surrounded myself with the people who were going to kind of be on my team. . .I think, in becoming a mother, just having some people that have got you back is great.

*Focus Group 1, mother raising children*

## Functioning

Most participants agreed that the competing demands of motherhood have an impact on mental health. Emotional wellbeing was related to feeling in control, productive and able to function daily:

I guess [emotional wellbeing] is productivity, doing things for myself as well. I went to the gym this morning and done all that, so that’s good. . . I feel more at ease when things are being done, when I am productive with my time, and then I’m able to be more present for my kids as well.

*Participant 6, mother raising children*

## Theme 2: Emotional wellbeing is something we feel

Women conceptualized emotional wellbeing to be a feeling. Two sub-themes describe their perspectives: Not mentally ill and Instinct.

## Not mentally ill

Most participants described recognizing emotional wellbeing in the absence of mental illnesses including anxiety and depression, however, one participant described her realization that this may not be an adequate description:

Well, not anxious, probably not depressed but now I am saying that to you, just the absence of illness sort of thing doesn’t quite cut it. [defining emotional wellbeing] That is a tricky question.

*Participant 5, mother raising children*

An opposing perspective was offered by an Aboriginal mother who described her innate knowledge of emotional wellbeing having not previously experienced mental illness in her life:

I feel [emotional wellbeing] inside definitely. . .it’s an interesting question, I guess it is strong. It is loud. Yeah, I can’t describe it any other way, but its [feels] just like a fire inside.

*Participant 8, mother raising children*

## Instinct

Participants who were not yet mothers recalled examples of becoming aware of emotional wellbeing in situations where mental health had changed:

That’s a tough question. . .I feel like I’m more conscious of when I’m not in a good space mentally. . .it is hard for me to recall when I have consciously thought to myself that I am emotionally well.

*Participant 11, pregnant woman*

For me, it is more obvious when I’m not in a good space. . .I can be tracking along normally when things are not challenging, but I am not consciously thinking I am in a good place until something throws me into a tizz

*Participant 10, woman planning pregnancy*

However, some participants raising children described instinctive awareness of emotional wellbeing:

I think we [learn as mothers] to trust our intuition with our babies. We need to trust our intuition with ourselves. . .isn’t it funny that it is hard to do that?

*Participant 2, mother raising a newborn*

Do what your eyes see and do what your heart says. . .belief in yourself as a mother comes from here [points to her heart]. If we are listening to our hearts in everyday mothering, then we have emotional wellbeing.

*Participant 8, mother raising children*

## Theme 3: The impact of environment

Women discussed barriers to optimizing emotional wellbeing, impacted by the environment they engaged with. Three sub-themes describe their perspectives: Social Media, Partner and Family and the Healthcare System.

## Social media

Despite its popularity, most women described negative, unwanted experiences of engaging with social media across the perinatal period. A phrase “doom scrolling” was used in relation to postpartum experiences of early morning awakening and the negative impact of online content.

I was going to say [emotional wellbeing] was just linked to social media and the pressure we put on ourselves by doom scrolling Instagram at 3:00 AM. Ever since I was pregnant, I looked at like *one* baby thing on Instagram. . . now just bam, bam. . .all my reels are newborn sleep routines and the perfect mothers.

*Focus Group 2, mother of one*

## Partner and family

The impact of partner and family members was described by participants who were pregnant or raising children and by participants planning pregnancy less so. The perceived societal view of the challenges mothers will face and the socially accepted impact on emotional wellbeing was shared by most participants:

I think it’s more like it’s your job. You know you’re a mum; you just do it. Like that’s kind of the view from society. And it’s something you signed up to do, so you know if it’s hard. . .I guess from a mum’s perspective, you can feel shame talking about it. . .I did sign up for this and I might not be coping with [motherhood]. . .Yeah, it’s not fair at all. Like it really is sh*t for mums

*Participant 6, mother raising children*

## Healthcare system

Access to maternity care is a unique experience within healthcare at a time where women are generally well. Women described barriers within primary care with no priority given to optimizing emotional wellbeing:

No. It was never like checked up or supported, encouraged or anything suggested. . . I think it would have made a huge difference, particularly going into birth.

*Participant 7, mother raising one child*

[Do you mean] in preparation for pregnancy specifically. . .then no is the short answer. . .. I did the EPDS [Edinburgh Postnatal Depression Scale] score with my midwife on our first visit. . .but I just feel kind of like I didn’t get what I needed [from the midwife] no.

*Participant 11, pregnant woman*

Current statistics estimate one in every 16 Australian child is born using assisted reproductive technology^35^ and the reported impact on mental health.^36^ One participant spoke of her journey to motherhood through in vitro fertilization (IVF) recognizing this would have been an ideal time to have considered optimizing emotional wellbeing.

No, no, no. I think we had the EPDS screening. . .we went through IVF, so it was highly medicalized. We’d had a counseling session because it was a donor conception situation. . .but nothing around sort of emotional preparedness or anything no

*Participant 5, mother raising children*

 Continuity of Care is considered optimal for aboriginal mothers^37^ offering a known healthcare provider throughout the perinatal period. One mother described her experience whilst receiving maternity care in a private hospital.

I would get a questionnaire handed to me over reception before I went in [to see the Dr] . . .it was quite basic, circle the smiley or the sad face. . .but the midwife or the OB never spoke to me about my answers. . .quite shocking really. . .It is far too casual and not a priority

*Participant 8, mother raising children*

## Theme 4: What women want

To explore women’s’ perceptions of potential solutions to the barriers experienced across the perinatal period, three sub-themes describe what women want if they are to be able to optimize mental health and emotional wellbeing: Continuity of Care; Tune in and Community.

## Continuity of care

Midwife means “with woman” and the partnership approach of midwifery is a vital component of professional practice optimizing maternal and neonatal outcomes.^31^ Most participants who had received continuity of care from a midwife were aware of the benefits of care delivered by a known midwife, but not all had accessed this model of care:

If I had a magic wand, I would provide continuity of midwifery care to everyone from conception. . . The midwives, they’re the experts, own everything in this space, like normal birth, normal delivery, health and Wellness. We don’t see a midwife until you’re nearly 20 weeks. We need continuity. My magic wand would be continuity.

*Focus Group 2, mother raising children*

## Tune In

The recognition of women being encouraged to trust their instincts was described by most participants recommending women in pregnancy be encouraged to “tune in” long before she becomes a mother if she is to optimize emotional wellbeing:

I think it’s hard to really say truly how we feel. I think [mums] are good at saying what the professionals want to hear. . . If we were kind to ourselves and tune in to kind of figure out what it is that we really need and communicate [our instincts] if we’re able [to do that], that will set us up [to be emotionally well].

*Focus Group 1, mother raising one child*

I guess just to be yourself and to trust in your own instincts if you know what I mean. It can’t be taught. It comes from within. Like it’s just there. Yeah, just believe in yourself. Like you can do it. Yeah, it comes from within.

*Participant 8, mother raising children*

## Community

The relationships around women during the perinatal period were described to be influential, having an impact on emotional wellbeing. Women described a desire that the relationships, both intimate and platonic, be prioritized and knowledge of emotional wellbeing be shared knowledge to achieve ideal outcomes:

One of the things I wish with my magic wand is, I want women to be taught to prioritize their community and relationships to the same extent. I think we prioritize romantic love so much, but we need to prioritize it in the same way within our community and our platonic friendships. . .It’s our collective knowledge, that’s the support that I got from my community. The healthcare providers can take care of my health, that’s great, but my community took care of me

*Focus Group 1, mother of multiple children*

## Theme 5: Perinatal mental health literacy

When discussing potential solutions to optimizing emotional wellbeing across the perinatal period, women described challenges within Perinatal Mental Health Literacy. Two sub-themes describe their perspectives: Missing Vocabulary and Partner and Family.

## Missing vocabulary

During both interview and focus group discussions, women experienced difficulty in accessing vocabulary to communicate effectively their thoughts in response to questioning suggesting the concept of emotional wellbeing may require further individual understanding across the perinatal period:

That’s a tricky question because no-one asked me about improving my mental health in pregnancy. I don’t think about it now, I don’t know what to say really. . .that is probably not a very helpful answer

*Participant 5, mother of one child*

I’ve never even contemplated that question. What would it take to be well on the journey to parenthood. Never. Because all my experience was about a schedule and maintaining a focus on the health of the baby.

*Focus Group 1, mother raising children*

## Partner and family

Within the focus group discussion, shared experience of the need to improve perinatal mental health literacy in non-birthing partners was described as an important direction for change:

For me, if I had a magic wand, I hate to say it because I feel like it’s a pipe dream, but if I had a magic wand, I’d want my husband to be me for a week. A whole week. So, I feel like he would truly get that understanding [of emotional wellbeing] . . .I just would love for him to not just say the words but to really feel it.

*Focus Group 1, mother raising children*

## Discussion

This study explored Australian women’s perceptions of emotional wellbeing, their experiences of perceived barriers in primary maternity care and their perspectives on possible solutions to promote emotional wellbeing in the perinatal period. To our knowledge, this is the first study to qualitatively explore and report this in Australia and internationally. The findings highlight several aspects of this experience for further discussion.

### Perinatal mental health literacy impacts recognition of emotional wellbeing

Our findings indicate Australian women lack proficiency in forming vocabulary around what it means to be emotionally well. Participants described a multidimensional experience based on affect and states of behavior that are recognized in the absence of mental illness. Poor mental health literacy among Australian women has been reported in research^38^ with Australian women. However, proficiency in communicating knowledge of mental health is reported in relation to identifying mental illness and difficulties including anxiety and depression. Theoretical discussions around conceptualizing perinatal emotional wellbeing^17,19^ reinforce the absence of broad understanding in this area, the complex and multidimensional construct of the experience and the orientation within academic literature toward mental illness when aiming to report on mental health. The extent to which Australian women possess the skills to self-identify emotional wellbeing and effectively communicate with primary maternity care providers is currently unknown and worthy of further exploration to enable promotion within the perinatal period.

In the absence of clear definitions or substantial theoretical models, the experience of Australian women across the perinatal period is heavily influenced by social and environmental factors including immediate family members, maternity healthcare providers, and the orientation of the healthcare system influenced by national perinatal health guidelines that lack explicit mental health promotion guidance.^3^ Women in this study described experiences of partners, immediate family and maternity healthcare providers unwilling to discuss mental health beyond the identification of depression or anxiety. This is supported by similar research findings illustrating the barrier of poor mental health literacy within the Australian population^38,39^ and within the midwifery profession.^40^ However, inconsistencies emerged between women who did not identify as Aboriginal Australian and those who did. Awareness and ability to articulate, identify and communicate emotional wellbeing did not rely on an academic definition or theoretical model but instinct and connection to an innate sense of being a woman before becoming a mother. Further research into improving perinatal mental health literacy among immediate family, primary maternity care providers and health systems is warranted to better understand how this will enable pregnant women and mothers to optimize emotional wellbeing.

While global health definitions^1^ emphasize physical, mental, and social wellbeing—not merely the absence of disease—Australian maternity care largely operates within a medical model focused on detecting illness.^25^ There is no standardized approach to promoting emotional wellbeing, and mental health is typically addressed only through risk-based screening for mental illness. This limited scope may contribute to poor perinatal mental health literacy which may negatively impacting ad-hoc mental health promotion opportunities, since there are no known antenatal interventions available to women accessing routine maternity care in Australia.^41^ Women in this study expressed support for shifting toward practices that prioritize emotional wellbeing during pregnancy, with the potential to improve mental health before the competing demands of motherhood. To support such changes, further engagement with both maternity care consumers and stakeholders is needed to identify needs, preferences, and perceived barriers within the current system.

### Releasing women’s potential

Most women in this study described a retrospective awareness of what may contribute to being able to realize optimal emotional wellbeing across the perinatal period. Through discussion, these women were able to identify where they may experience wellbeing in the absence of mental illness. Most women discussed the influence of immediate family members and described the dynamic influences they have on achieving optimal levels of emotional wellbeing. Bronfenbrenner’s ecological model^42^ is a framework that can be incorporated to understand the complex interactions that influence human experience. A complete model for this study is shown in Supplemental Figure 2 (Supplemental File 4). The interpersonal relationship(s) across the perinatal period extends beyond the immediate family to include primary maternity care providers including midwives, GP, obstetricians, and maternal child health nurses. If women are to begin to reach their potential, this will be influenced by all levels.

Pregnant women are thought to have increased motivation to adopt behavior change toward better health outcomes.^43^ It is proposed that the potential to optimize emotional wellbeing is unrealized. However, pregnant women and mothers can leverage the uniqueness of the perinatal period, which includes increased clinical contact with primary maternity care providers. All participants, regardless of Aboriginality or history of mental illness described a desire to access standardized conversations within primary care focused on optimizing emotional wellbeing to support a positive transition to motherhood. In maternity healthcare, the orientation toward safeguarding neonatal outcomes and optimizing maternal physical health overshadows the promotion of maternal mental health and emotional wellbeing. Women in this study shared experiences of accessing medicalized models of care with attention largely directed toward fetal health and wellbeing, labor and birth outcomes, and maternal physical health. This imbalance risks reinforcing the perception that maternal mental health and wellbeing is secondary, despite evidence that emotional health during pregnancy is integral to both maternal and neonatal outcomes. As shown in the ecological model (Supplemental File 4) women and mothers with improved perinatal mental health promotion literacy and ownership of her emotional wellbeing as a woman, it is proposed that her potential could be realized within a system orientated to nurture and promote the individual nature of emotional wellbeing.

### Preparing midwives to optimize perinatal emotional wellbeing

All women in the study valued the role of the midwife and spoke positively about receiving midwifery care. However, no examples were given of optimizing emotional wellbeing within midwifery care. One participant described her awareness of the midwife’s presence during postnatal hospital care and her willingness to engage in everyday conversation and although not explicitly communicated, the midwife presence was interpreted as an interest in evaluating postnatal wellbeing. Women had a good understanding of continuity of midwifery care models and the benefits of getting to form a trusting relationship with a midwife through pregnancy. However, not all participants had accessed this model of care, yet there was a unanimous acknowledgment that midwives are best placed to provide this opportunity. The contemporary midwife is educated and trained to care for women underpinned by a philosophical view that prioritizes a trusting professional relationship with women.^44^ The potential for midwives to optimize perinatal emotional wellbeing is supported by similar research findings demonstrating the health promotion capabilities of midwives.^45,46^ Preparing the midwifery profession to work to a mental health promotion scope will be influenced by reported barriers including lack of confidence, knowledge, and existing organizational priorities.^47^ Although health promotion is acknowledged as core midwifery scope, mental health promotion is much less reported within the midwifery scope of practice.^48,49^ The potential to utilize the capacity of the profession is unrealized in Australia. Changes in midwifery practice to include mental health promotion could play a role in strengthening population health outcomes. A summary of recommendations from this study is displayed in Table 2.

**Table 2.** Study recommendations.

Level	Recommendations
Pregnant Women & Mothers	• Require knowledge, awareness and proficiency of language to self-identify and communicate what it means to be emotional well at individual levels and effectively communicate identified needs with partners, friends, family and maternity healthcare providers. • Knowledge, awareness and confidence to prioritize and incorporate self-identified experience to optimize levels of emotional wellbeing into daily routines in the perinatal period.
Midwifery Workforce	• Changes to current midwifery practice to include facilitating access to mental health promotion opportunities for pregnant women and mothers may unlock improvements in emotional wellbeing for women.
Maternity Healthcare Systems	• A change in current practice to include screening for emotional wellbeing in pregnancy to create opportunities to recognize and improve mental health in pregnancy • Engage consumers of maternity healthcare to explore their perspectives on solutions to current practice that would best meet their maternal mental health promotion needs. • Engagement with providers of maternity healthcare should occur alongside consumers to capture perspectives of perceived challenges to be overcome in future projects.
Perinatal Mental Health Policy	• Integrating the promotion of mental health and emotional wellbeing into existing perinatal mental health policies shifts the focus from solely preventing or treating mental illness to proactively addressing how pregnant women and mothers can strengthen mental health and wellbeing.

### Strengths and limitations

We believe this study is the first to use qualitative methodology to explore Australian women’s perspectives on perinatal emotional wellbeing. Several participants in both interviews and focus groups were from the state of Western Australia and brought a variety of experiences, including perspectives related to Aboriginality, preconception pregnancy planning, in vitro fertilization, pregnancy, and raising multiple children. The sample included a relatively even spread between women who had previously experienced mental illness and those who had not. Limitations of the study include that all participants spoke English as their first language; findings may differ in populations who speak languages other than English at home. Not all states and territories were represented; however, we acknowledge representativeness is not the goal of qualitative research. Most participants were raising children and were at a different stage of the perinatal period than those planning pregnancy or currently pregnant. Therefore, recall bias may have influenced the perspectives shared by mothers who were asked to reflect on their experiences during the interview or focus group. Finally, the demographics included provide an opportunity to consider the transferability of these findings to populations outside of the Australian context.

## Conclusions

This study provides new insight into how Australian women understand emotional wellbeing during the perinatal period. Findings suggest that women largely define emotional wellbeing by the absence of illness, and it is multidimensional—shaped by environmental and relational influences. While the midwifery profession and continuity models of midwifery care were highly valued, participants highlighted limited or no opportunities to promote mental health and emotional wellbeing within routine care, which remains orientated toward detecting risk or presence of mental illness. Strengthening perinatal mental health literacy among women, families, and care providers may help women recognize and express their individual needs, thereby building confidence to strengthen mental health through the perinatal period. Further research is warranted to explore the impact of improved knowledge and mental health literacy in perinatal settings across Australia.

## Supplemental Material

sj-docx-1-phj-10.1177_22799036251395270 – Supplemental material for Exploring emotional wellbeing in the perinatal period: A qualitative study in Australia Supplemental material, sj-docx-1-phj-10.1177_22799036251395270 for Exploring emotional wellbeing in the perinatal period: A qualitative study in Australia by Lesley Pascuzzi, Karen Heslop, Helen Skouteris and Zoe Bradfield in Journal of Public Health Research

sj-docx-2-phj-10.1177_22799036251395270 – Supplemental material for Exploring emotional wellbeing in the perinatal period: A qualitative study in Australia Supplemental material, sj-docx-2-phj-10.1177_22799036251395270 for Exploring emotional wellbeing in the perinatal period: A qualitative study in Australia by Lesley Pascuzzi, Karen Heslop, Helen Skouteris and Zoe Bradfield in Journal of Public Health Research

sj-docx-3-phj-10.1177_22799036251395270 – Supplemental material for Exploring emotional wellbeing in the perinatal period: A qualitative study in Australia Supplemental material, sj-docx-3-phj-10.1177_22799036251395270 for Exploring emotional wellbeing in the perinatal period: A qualitative study in Australia by Lesley Pascuzzi, Karen Heslop, Helen Skouteris and Zoe Bradfield in Journal of Public Health Research

sj-docx-4-phj-10.1177_22799036251395270 – Supplemental material for Exploring emotional wellbeing in the perinatal period: A qualitative study in Australia Supplemental material, sj-docx-4-phj-10.1177_22799036251395270 for Exploring emotional wellbeing in the perinatal period: A qualitative study in Australia by Lesley Pascuzzi, Karen Heslop, Helen Skouteris and Zoe Bradfield in Journal of Public Health Research
