# Supplementary material for: Exploring emotional wellbeing in the perinatal period: A qualitative study in Australia
Source: J Public Health Res. 2025 Nov 13;14(4):22799036251395270. doi: 10.1177/22799036251395270 (PMC12615929; doi:10.1177/22799036251395270)
Supplement: sj-docx-1-phj-10.1177_22799036251395270 – Supplemental material for Exploring emotional wellbeing in the perinatal period: A qualitative study in Australia [file sj-docx-1-phj-10.1177_22799036251395270.docx]

Supplementary File 1

Interview Guide – Australian Women

1. Can you describe to me what you think it means to be emotionally well?
   - 1. Prompt – can you tell me about the most recent day you have felt like this
     2. Prompt – can you describe what you mean by not being ill?
     3. Prompt – if I was to watch you as an emotionally well woman, what would I see you doing/saying/feeling?
2. What, if any, experiences can you share to describe what was challenging or what made it easier to promote emotional wellbeing?
3. If you had a magic want, what solutions do you think could effectively support pregnant women and mothers towards optimal mental health and emotional wellbeing in the future?
