# Supplementary material for: Exploring emotional wellbeing in the perinatal period: A qualitative study in Australia
Source: J Public Health Res. 2025 Nov 13;14(4):22799036251395270. doi: 10.1177/22799036251395270 (PMC12615929; doi:10.1177/22799036251395270)
Supplement: sj-docx-2-phj-10.1177_22799036251395270 – Supplemental material for Exploring emotional wellbeing in the perinatal period: A qualitative study in Australia [file sj-docx-2-phj-10.1177_22799036251395270.docx]

Supplementary File 2

Focus Group Guide – Australian Women

1. Can you describe to me what you think it means to be emotionally well on the journey through pregnancy to parenthood?
2. Please describe any experience(s) with a midwife or perinatal mental health professional during pregnancy or parenthood where meaningful mental health promoting conversations had an impact on emotional wellbeing or discuss any challenges you experienced.
3. If you had a magic wand, what solutions can you identify as possible ways to improve the experiences of pregnant women and mothers in the future.
