# Supplementary material for: Exploring emotional wellbeing in the perinatal period: A qualitative study in Australia
Source: J Public Health Res. 2025 Nov 13;14(4):22799036251395270. doi: 10.1177/22799036251395270 (PMC12615929; doi:10.1177/22799036251395270)
Supplement: sj-docx-3-phj-10.1177_22799036251395270 – Supplemental material for Exploring emotional wellbeing in the perinatal period: A qualitative study in Australia [file sj-docx-3-phj-10.1177_22799036251395270.docx]

Supplementary File 3 – Table 3 – Supporting quotations from interview and focus group transcripts

| **Theme** | **Sub-theme** | **Quotation** |
| --- | --- | --- |
| Emotional Wellbeing is a state of “being” | Energy                                                  Support                                    Functioning | “If I'm eating better and sleeping better and able to regulate all those things, this allows me to be more [emotionally] well. I've never really been one for employing strategies such as mindfulness or meditation or anything like that really. I struggle to quieten my brain and be still in those moments whenever I do meditation or mindfulness on my own. But when it is facilitated by other people, I find them incredibly helpful, but I always struggle to initiate those things myself. After pregnancy, when I was mothering, probably nine months postnatal, I used exercise as a big strategy to help my wellbeing. That was helpful at the time.”  *P7, mother raising one child*    “I completely agree with you, 100% on the sleep exhaustion.  My daughter is two weeks old today and yeah, for the last 2 nights she's only slept on someone, so I was praying at five o’clock this morning for her to sleep so I could sleep. I think [sleep deprivation] kind of taints your perception of everything, literally because you're so tired. One thing could happen, and [I think] “oh my God, I'm failing”  *Focus Group 2, early postpartum mother*    I had my mum, who actually has a midwifery background as well, so she has more experience than most. She was able to talk me through it [what to expect], but I don't know how that experience would have gone if I didn't have her, those safe people that I had a really good relationship with that I could go and to and say actually I want to know about this or this is worrying me...If I didn't have these people to reassure me, I don't think I would have been ok.  *Focus Group 1, mother raising one child*      One of my biggest strategies [for emotional wellbeing] is to feel supported. Engaging support from the people in my life. So, my husband, my parents, my friends, especially now being a mother, I'm enlisting their support in parenting too. I bought in the people, my mum or my mother-in-law or, you know, my closest friend, people who would be that proxy I guess parenting my son when I couldn't [because of mental illness]. But had I not had the insight and the ability to do that, I wouldn't be confident to say that I was doing a good job as a mum.  *P7; mother raising one child*    “I know that I can reach out to my husband, my family and my support circle but I probably didn't prepare myself as much as I probably should have during pregnancy the first time...in saying that I did use my networks and my circles, and I guess my maturity in my second pregnancy. I chatted to friends and family that had gone through it before, so in terms of that, [I had] those tools in my toolkit. I leant on family and friends for their knowledge and their experience in going through pregnancy and motherhood. I definitely used the people around me. So, in that sense, yeah that kind of kept me on track with [emotional wellbeing] .... I know that my emotional well-being is catered for and then I know that I have somewhere to go for it. I don't feel lost.”  *P1, mother raising multiple children*    I think emotional well-being is, well, mainly, I was told [by a midwife] when I became a mum that it's OK if you need to just close the door on your crying baby and have a cry yourself. I think emotional well-being is if you don't need to do that. [that means] you're coping and it's not too hard and you've got a good amount of support around you and that if you are struggling that you had someone to help you in whatever way that is, maybe sharing the load or sharing your thoughts or something else. I think [emotional] wellbeing doesn't mean that you're well [all the time]. It just means that you are content and you're coping and you're not struggling, and you don't feel like you're drowning  *P3, mother raising one child*    **“**When you are emotionally well, you kind of [think] ok, that's fine, I can deal with [the day]. But then when you are less emotionally well, you just get a bit sort of deflated... that's when those things kind of make you [think] what is this, what's wrong with me, what is wrong with my parenting style? I think they're sort of signals [for me] to think “ok it's probably because I am tired, you're just not feeling on top of it, and all those sorts of things...I think it's just that being emotionally well; it's being able to cope but not just cope. I think when I'm doing it well, I'm doing it in the moment when I'm not doing it well, I'm doing it immediately after and taking that breath and thinking now I'm in the tantrum as well [as the kids].  *P5, mother raising multiple children* |
|  |  |  |
| Emotional Wellbeing is a something we “feel” | Not Mentally Ill                                              Instinct | I guess it won't be illness. It's wellness like you feel content.  I think [emotional wellbeing] is that you're not searching for something to make you happy, but you're not dwelling on things that made you sad like it is.  Just like, we've just got the thing to do, and I think motherhood definitely changes that for a lot of people.  *P3, mother raising one child*    “Umm. Not anxious, not depressed. Calm. Things are under control. Feeling supported.  Yeah, they're probably the top things that come to mind.”  *P2, mother raising a newborn*      Oh gosh, Lord, where would I even begin?  I think being emotionally, well, just in general, but certainly on the journey to parenthood, it's just around being calm content, not sort of feeling that anxiety, not feeling uncertainty [about the future], especially moving into first time parenthood is certainly normal and expected.  But I guess it's more about, you know, being comfortable with [uncertainty] and knowing you know where to find help if you need it.  And all those kinds of things, I think, all contribute to [emotional] wellbeing and certainly having support around you as well.  If you've got a solid support circle, that's a big helper in having emotional wellbeing.  *P10, mother raising multiple children*      You sort of feel, well I felt very bombarded with lots of different opinions coming from lots of different angles or not even opinions, just experiences and that can be very overwhelming. I think what helped me, I guess, stay grounded and find my sense of peace again was being able to come back to that place of safety both physically and emotionally as well. I think you know, in anything, when you're talking with medical professionals and when people can offer you that sense of space and the space to be able to process what you're thinking and feeling and allowing you to come back to that feeling of your centre, I guess.  *Focus Group 1, mother raising multiple children* |
| The Impact of Environment | Social media | I think it is about being connected intuitively. To what is our own intuition around mothering rather than what does the book say, what does the website say? What does social media say?  That way away from what does your heart say? What is in your soul and how does that speak to you? Because all of us mums have it in there, like every day could be just when you get up and you get in the kids ready for school today. And if it is in us, you just got to pick up the signs or believe in yourself. Those gut feelings. Your heart like all, just heart. It is there. And I think we don't listen to it enough. When there are books and social media, and all this lady is doing this with their baby and someone else is doing that.  I would say first “well what does your heart tell you?” Do what your eyes see and what do what your heart says”. It is that clear to me that [points to her heart] is where believe in yourself comes from. Yeah. And if we were listening to what your heart tells you in your everyday mothering, then I can feel emotionally well.  *P8, mother raising multiple children*    I found to help myself be emotionally well, I actually deleted social media off my phone for a long like it's back on there now, but I'm feeling like I need to get it off again and that was a way that I could self-check myself that that I was doom scrolling and that it was wasn't making me feel nice. It didn't make me feel good, so by deleting it off I still had it my computer, but I found that deleting it off my phone meant that I wasn't tempted to do that 3:00 AM doom scroll.  *Focus Group 2, mother raising multiple children* |
|  | Partner & Family | **“**I'll just chip in there and mirror what was said and support everything that she said The expectation that you set on yourself and the expectation that is set external from your own your own small circle, your partner, your family is so important in this journey to recognize that the expectations shouldn't be as high as their potentially being set by yourself or externally and being able to rely on that support but also support yourself and remembering that that's one of the most important things that you do is that support for yourself first and then hopefully it's coming from others around you as well.  *Focus Group 1, mother raising one child*    My husband worked FiFo [when motherhood started]. He was away for, you know, up to two or three months at a time. And I had no family here, so I was literally on my own. And I remember none of my friends had kids yet. No one understood. So, when you talk about support, I think support is from people who have an understanding about what's going on as well.  *Focus Group 2, mother raising multiple children*    “As soon as [family] finds out you're pregnant, they want to tell you their story and that can be positive or negative depending on their own experiences. You sort of feel, well I felt very bombarded with lots of different opinions coming from lots of different angles or not even opinions, just experiences and that can be very overwhelming.”  *Focus Group 1, mother raising multiple children* |
|  | Healthcare System | I've got to be honest, it's probably not a great reflection about health system, but I don't think that's sort of come up at all really.  Even my GP, who obviously you know I do a mental health plan every year, so he's very aware of my mental health history.  And you know my diagnosis and all that kind of thing.  He's also aware that I've been doing IVF and that I've been doing it as a solo journey and you know I've not really thought about it now until you asked the question, but I've just realized he's not actually flagged [promoting mental health] at any point, [saying] hey, you know, we probably need to have a conversation about how we're going to manage [your] mental health throughout this journey.  Sorry. Yeah, that's really interesting because it's not, it's not really come up for me at all.  *P10, preconception woman planning pregnancy*    As soon as you asked us that question, I thought, I don't remember a single time when somebody actively looked after my wellbeing. [Midwives] give you a questionnaire or something like that.  They picked up on a sign or a symptom, that maybe you're a bit emotionally unwell then it was straight to the psychologist with you, but not once did anybody ever kind of say to me “What does it mean for you to be well, to be prepared?  [As a result] through my own advocacy and my own finding the team around me that I wanted throughout pregnancy I could try and support myself.  *Focus Group 1, mother raising multiple children*    I was referring myself [to the mental health team] because I wanted to set myself up with a support system for after the baby's born... to prepare and forward plan... [the experience] kind of like I defeated the purpose of going in the first place. I feel like we could have just gone in with the main thing. I wanted to forward plan. That was that I felt was my aim.  I felt like it was very, what's the word? I guess messy...... I feel like he really didn't know what to do with me. I told him, I was trying to set myself up for postpartum, so that's why I'd gone there, and I guess he just didn't really know what to do with that...before I left, he offered me medication and then said, “Do you need to see us again?  And I was like, I don't think so. I don't even know what I got out of that...And then I went home. I just went in the hopes that maybe I would learn something new, that they would have tips for me, or they would. I don't know. That's why I went. I just left deflated, cried and didn’t really get what I went for.”  *P11, first time pregnant woman* |
|  |  |  |
| What Women Want | Continuity of Care                          Tune In                                      Community | I guess it would be a midwife, I guess if every woman had continuity, you know, like had their own midwife, somebody who knew them, who they connected with and there was a good rapport, someone who understood if they had mental health history, who understood that they were struggling with different aspects or, you know, who could help with all those aspects and refer on the conduct screenings.  *P7, mother raising one child*    I think I love the idea of continuity, not necessarily all the way through, but if you are having fertility issues you get sent to a fertility coordinator and that's your coordinator until you've either decided it's not for you or you've got pregnant and you're 12 weeks [before] you can go to midwife...  Ideally, you know you're early pregnant and maybe there's a fertility specialist and an early pregnancy specialist and then your midwife. And so that if you have any questions, you know who to go to like this, you've got a named contact there for every stage.  *Focus Group 1, mother raising one child*    I think we need to be honest with ourselves, in the face of others, you know. I think if we really tuned into what we need and like you said, like give voice to that and trust it without the judgment that we get from external people and we hold strong to that.  It's really hard to articulate that and communicate that and for that to be received and respected, but that's a little bit of a big dream I think about, you know, if we really were, if we really said it like it was for us and I'm not saying we're not doing that. But I think it's hard. I think it's hard to really say it like it is and say it truly how we feel.  I think we're really good at kind of saying what the professionals want to hear and we're not necessarily aware of that. I think you know, on reflection we, we're like, oh, crap, I wish I'd said that like I wish I actually made my point. If we're kind to ourselves and Tune In to kind of figure out what it is that we really need and communicate that, if we're if we're able, that will set us up well.  *Focus Group 1, mother raising multiple children*    Yeah, it's there. Comes from inside. Yep. I'm not at all influenced from the outside. I'm not at all. I think I learnt that with my first-born [son]. Listening to all that noise. It was actually making me feel like “Oh my gosh, I'm not doing good enough”. And then something clicked, and I said “no, listen.” I just let all that go and started letting [my mothering instinct] shine from me. [Now] I look back... my eldest is 22 this year and my youngest not even a year old. I'm told by everyone how beautiful all my kids are. And so, I'm doing it. Doing what grandma did. Doing what my mum did. From here [points to her heart].  *P8, mother raising multiple children*  [With a magic wand} I would probably want to connect with [pregnant women] before she had a child. That would be one thing...I think because I think it's too late to prepare yourself and [emotional wellbeing] once you've had your baby because you're already in [mothering]. Once the baby arrives, you're in this state where you're thinking about them every single minute of every day and not yourself anymore. So, I think you need to prepare yourself as much as you can for what may happen to [mental health and emotional wellbeing] post when the baby arrives, because you'll be tending to their every need and suddenly it could creep up on you. So, I think that preparation beforehand would be my idea.  *P1, mother raising multiple children* |
|  |  |  |
| Perinatal Mental Health Literacy | Missing Vocabulary                            Partner & Family | [When] I'm not having poor mental health day or sad day, it's a good day, but I don't think to myself.  “Oh, it could be better because it's already a good day [not to have mental illness]”. I don’t do that, no. I don’t want to boost myself because I feel as good as I can just without depression or anxiety.  There's nothing in me saying today could be better. I'm just grateful for the things that I do have and so yeah, that's I guess something that I've learned when I was trying to self-manage my depression and anxiety and teaching myself to meditate.  A lot of it is just gratitude. I can’t describe it any other way.  *P11, first time pregnant woman*    A view that I think is out there, but there's been a few things like. And someone said, oh, you know how you are doing? And I'm like, oh, it's been pretty shit. And they're like, oh, well, that's well, that's parenting, isn't it?  OK. Yeah, that's what it is. Oh, you know, oh, you're exhausted now. You wait till that baby comes. It's just like when people say it to me, it's just like, awful. You just think shut up, walk away. But, you know, it's just how to navigate that rather than all things are probably going to get worse. So, buckle in.  *P6, mother raising multiple children*  Probably make sure that she's OK.  That is one of the things my mum kept saying to me – was I happy?  Mum happy, happy bub.  Even now, I still think of that as if I'm not coping that the house falls apart, even though my husband is here, he's probably next to useless for that kind of thing.  Ohm, he falls apart when I fall apart.  So that's not good for [emotional wellbeing].  *P3, mother raising one child* |
|  |  |  |
