# Supplementary figures and images for: Exploring emotional wellbeing in the perinatal period: A qualitative study in Australia
Source: J Public Health Res. 2025 Nov 13;14(4):22799036251395270. doi: 10.1177/22799036251395270 (PMC12615929; doi:10.1177/22799036251395270)

Supplementary File 4 – Figure 2 – Ecological model


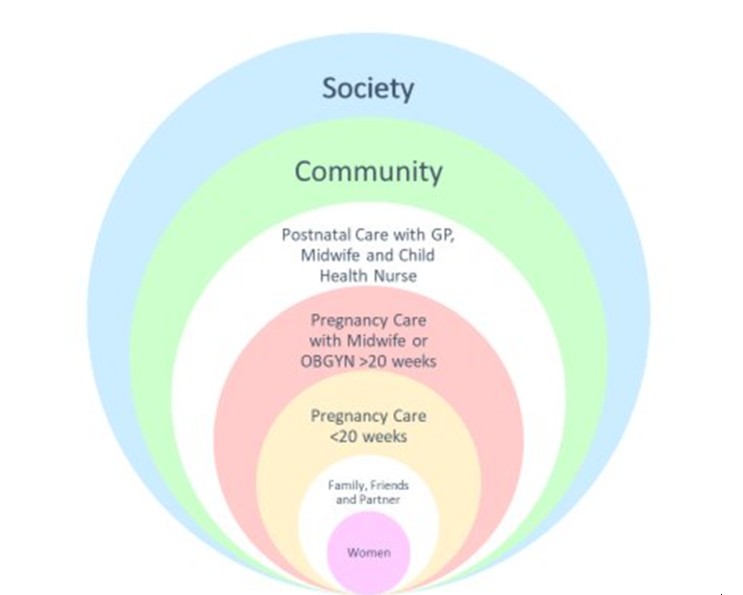

Supplement: sj-docx-4-phj-10.1177_22799036251395270 – Supplemental material for Exploring emotional wellbeing in the perinatal period: A qualitative study in Australia [file sj-docx-4-phj-10.1177_22799036251395270.docx]
